# Blunted apoptosis of erythrocytes in mice deficient in the heterotrimeric G-protein subunit Gαi2

**DOI:** 10.1038/srep30925

**Published:** 2016-08-08

**Authors:** Rosi Bissinger, Elisabeth Lang, Mehrdad Ghashghaeinia, Yogesh Singh, Christine Zelenak, Birgit Fehrenbacher, Sabina Honisch, Hong Chen, Hajar Fakhri, Anja T. Umbach, Guilai Liu, Rexhep Rexhepaj, Guoxing Liu, Martin Schaller, Andreas F. Mack, Adrian Lupescu, Lutz Birnbaumer, Florian Lang, Syed M. Qadri

**Affiliations:** 1 Institute of Cardiology, Vascular Medicine and Physiology, University of Tuebingen, Germany; 2 Department of Gastroenterology, Hepatology and Infectious Diseases, University of Duesseldorf, Germany; 3 Department of Internal Medicine, Charité Medical University, Berlin, Germany; 4 Department of Dermatology, University of Tuebingen, Germany; 5 Institute of Biochemistry and Molecular Biology, University of Bonn, Germany; 6 Institute of Anatomy, University of Tuebingen, Germany; 7 Neurobiology Laboratory, National Institute of Environmental Health Sciences, National Institutes of Health, Research Triangle Park, Durham, NC, USA; 8 Institute of Biomedical Research (BIOMED), School of Medical Sciences, Catholic University of Argentina, Buenos Aires, Argentina; 9 Department of Pathology and Molecular Medicine, McMaster University, Hamilton, ON, Canada

## Abstract

Putative functions of the heterotrimeric G-protein subunit Gαi2-dependent signaling include ion channel regulation, cell differentiation, proliferation and apoptosis. Erythrocytes may, similar to apoptosis of nucleated cells, undergo eryptosis, characterized by cell shrinkage and cell membrane scrambling with phosphatidylserine (PS) exposure. Eryptosis may be triggered by increased cytosolic Ca^2+^ activity and ceramide. In the present study, we show that Gαi2 is expressed in both murine and human erythrocytes and further examined the survival of erythrocytes drawn from Gαi2-deficient mice (*Gαi2*^−/−^) and corresponding wild-type mice (*Gαi2*^+/+^). Our data show that plasma erythropoietin levels, erythrocyte maturation markers, erythrocyte counts, hematocrit and hemoglobin concentration were similar in *Gαi2*^−/−^ and *Gαi2*^+/+^ mice but the mean corpuscular volume was significantly larger in *Gαi2*^−/−^ mice. Spontaneous PS exposure of circulating *Gαi2*^−/−^ erythrocytes was significantly lower than that of circulating *Gαi2*^+/+^ erythrocytes. PS exposure was significantly lower in *Gαi2*^−/−^ than in *Gαi2*^+/+^ erythrocytes following *ex vivo* exposure to hyperosmotic shock, bacterial sphingomyelinase or C6 ceramide. Erythrocyte Gαi2 deficiency further attenuated hyperosmotic shock-induced increase of cytosolic Ca^2+^ activity and cell shrinkage. Moreover, *Gαi2*^−/−^ erythrocytes were more resistant to osmosensitive hemolysis as compared to *Gαi2*^+/+^ erythrocytes. In conclusion, Gαi2 deficiency in erythrocytes confers partial protection against suicidal cell death.

G protein-coupled receptors activate heterotrimeric G proteins via ligand binding, thereby modulating the activity of cellular effectors and consequently regulating a wide array of cell functions^1^,^2^. The putative function of the functional class of G protein Gαi is defined by their ability to downregulate cAMP levels by inhibition of adenylyl cyclase^2^,^3^. The closely-related Gα members Gαi1, Gαi2, and Gαi3, sharing 85–95% of their amino acid sequence identity, are characterized by their sensitivity to pertussis toxin^2^,^3^. Gαi2, the quantitatively predominant Gαi isoform, is a decisive regulator of leukocyte, endothelial and platelet functions^4^,^5^,^6^,^7^. Further putative roles of Gαi2 signaling include ion channel regulation, cell differentiation, proliferation and apoptosis^8^,^9^,^10^,^11^,^12^. Effector kinases of G-protein signaling include phosphoinositide 3-kinases^13^, which are known to be involved in the regulation of apoptosis^14^. Gαi2 further influences Ca^2+^ signaling in nucleated cells by the activation of TRPC4 channels which, in turn, increases Ca^2+^ influx^15^. In cardiomyocytes, Gαi2 has been shown to modulate the activity of L-type voltage-dependent Ca^2+^-channels^11^. Furthermore, Gαi2 is a powerful regulator of cytosolic Ca^2+^ activity in islet beta cells^12^ and neutrophils^16^, thus, regulating a variety of Ca^2+^-dependent cell functions. Phenotypically, Gαi2 knockout mice have been reported to display a predisposition towards a wide range of disorders including growth retardation, inflammatory bowel disease, carcinogenesis, cardiac arrhythmia and impaired haemostasis^4^,^17^,^18^.

Similar to nucleated cells, erythrocytes may undergo suicidal death or eryptosis^19^,^20^, which, similar to apoptosis, is triggered by osmotic shock and characterized by cell shrinkage and cell membrane scrambling^20^,^21^. Eryptosis may be triggered by activation of Ca^2+^-permeable cation channels^20^ which subsequently leads to increase of cytosolic Ca^2+^. The molecular identity of these cation channels has not been completely characterized but apparently involves TRPC6 channels^22^. The cation channels are activated by prostaglandin E_2_, which is formed following exposure of erythrocytes to hyperosmotic shock^19^. The channels are further activated by a wide variety of cell stressors, xenobiotics and endogenous mediators^19^. Ca^2+^ activates Ca^2+^-sensitive K^+^ channels with exit of KCl and osmotically obliged water and thus cell shrinkage^19^,^20^. An increase of cytosolic Ca^2+^ is further followed by stimulation of cell membrane scrambling with exposure of phosphatidylserine (PS) at the cell surface^19^,^20^. The Ca^2+^ sensitivity of cell membrane scrambling is further enhanced by ceramide^21^. PS-exposing cells are bound to macrophages, engulfed and degraded and thus cleared from circulating blood^19^,^20^,^23^,^24^,^25^. To the best of our knowledge, the impact of Gαi2 on erythrocyte survival and suicidal death has hitherto not been reported.

In the present study we explored whether the Gαi2 isoform is expressed in erythrocytes and whether it participates in the regulation of erythrocyte survival. To this end, eryptosis was determined in erythrocytes from Gαi2 knockout mice (*Gαi2*^−/−^) and their wild type littermates (*Gαi2*^+/+^).

## Results

**Figure 1:**
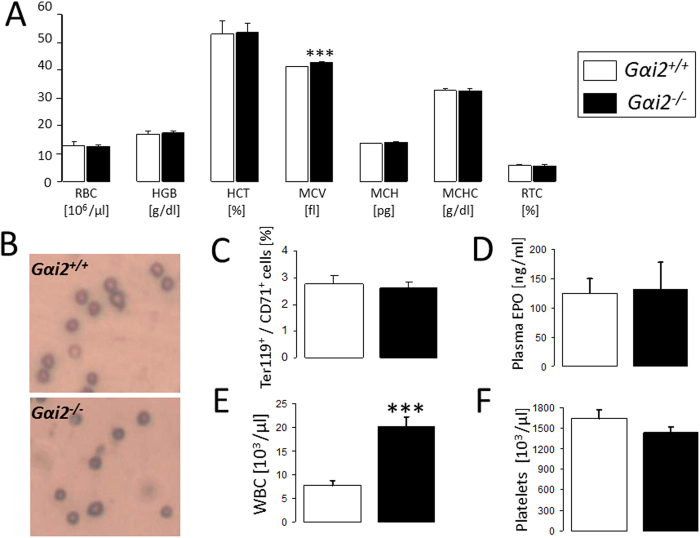
Blood parameters. Means ± SEM of erythrocyte count (RBC), haemoglobin concentration (HGB), haematocrit (HCT), mean corpuscular volume (MCV), mean corpuscular haemoglobin (MCH), mean corpuscular hemoglobin concentration (MCHC) and reticulocyte count (RTC) (**A**, n = 8), Ter119/CD71 positive cells (**C,** n = 6), plasma erythropoietin (EPO) levels (**D,** n = 3–4), leukocyte count (**E,** n = 8) and platelet count (**F,** n = 8) in *Gαi2*^+/+^ and *Gαi2*^−/−^ mice. ***(p < 0.001) significantly different from *Gαi2*^+/+^ mice. (**B**) May-Grünwald staining of erythrocytes from *Gαi2*^+/+^ and *Gαi2*^−/−^ mice.

The present study addressed the impact of Gαi2 on eryptosis in mice. To this end, experiments were performed in mice lacking functional Gαi2 (*Gαi2*^−/−^) and corresponding wild type mice (*Gαi2*^+/+^). As shown in Fig. 1A, erythrocyte count, hemoglobin concentration, hematocrit, mean corpuscular hemoglobin, mean corpuscular hemoglobin concentration and the percentage of reticulocytes were not significantly different between *Gαi2*^−/−^ and *Gαi2*^+/+^ mice. The mean corpuscular volume was slightly, but significantly larger in *Gαi2*^−/−^ than in *Gαi2*^+/+^ erythrocytes (41.1 ± 0.3 fl for *Gαi2*^+/+^ mice versus 42.8 ± 0.2 fl for *Gαi2*^−/−^ mice; n = 8, p < 0.001). *Gαi2*^−/−^ erythrocytes are, thus, normochromic and moderately larger as compared to *Gαi2*^+/+^ erythrocytes. May-Grünwald staining further revealed no apparent changes in erythrocyte shape from *Gαi2*^−/−^ mice as compared to erythrocytes from *Gαi2*^+/+^ mice (Fig. 1B). The percentages of CD71/Ter119 positive cells were similar in *Gαi2*^−/−^ and *Gαi2*^+/+^ mice suggesting similar patterns of dynamic erythrocyte maturation *in vivo* (Fig. 1C). Plasma erythropoietin concentrations were further similar in *Gαi2*^−/−^ and *Gαi2*^+/+^ mice (Fig. 1D). Consistent with a previous report^26^, we observed leukocytosis in *Gαi2*^−/−^ mice (Fig. 1E) which is attributed to increased production of proinflammatory cytokines in *Gαi2*^−/−^ mice^18^. The platelet count in *Gαi2*^−/−^ mice was, however, not significantly different from *Gαi2*^+/+^ mice (Fig. 1F).

**Figure 2:**
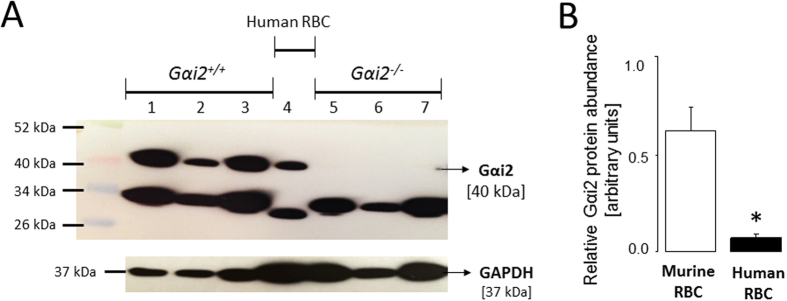
Gαi2 expression. (**A**) Western blots showing Gαi2 (40 kDa) and GAPDH (37 kDa) expression in erythrocytes from *Gαi2*^+/+^ (bands 1, 2 and 3) or *Gαi2*^−/−^ (bands 5, 6 and 7) mice and humans (band 4) in whole blood (bands 1 and 5), diluted whole blood (bands 2 and 6; 1:3.7 dilution) and purified erythrocytes (bands 3 and 7). (**B**) Means ± SEM of Gαi2 abundance in murine and human erythrocytes relative to the loading control GAPDH (n = 3).

Immunoblotting was employed to examine whether Gαi2 is expressed in human and murine erythrocytes. To this end, erythrocytes from humans or from mice were isolated and purified. Equal amounts of protein lysates were immunoblotted. GAPDH served as a loading control. As depicted in Fig. 2A, the incubation with Gαi2 specific antibodies yielded a single band of 40 kDa in human erythrocytes as well as erythrocytes from *Gαi2*^+/+^ mice, but not in erythrocytes from *Gαi2*^−/−^ mice. The bands appearing below 40 kDa are presumably the result of non-specific antibody binding. Densitometry analysis revealed that Gαi2 protein is significantly more abundant in mouse erythrocytes as compared to human erythrocytes (Fig. 2B). Thus, Gαi2 is expressed in both human and murine erythrocytes.

**Figure 3:**
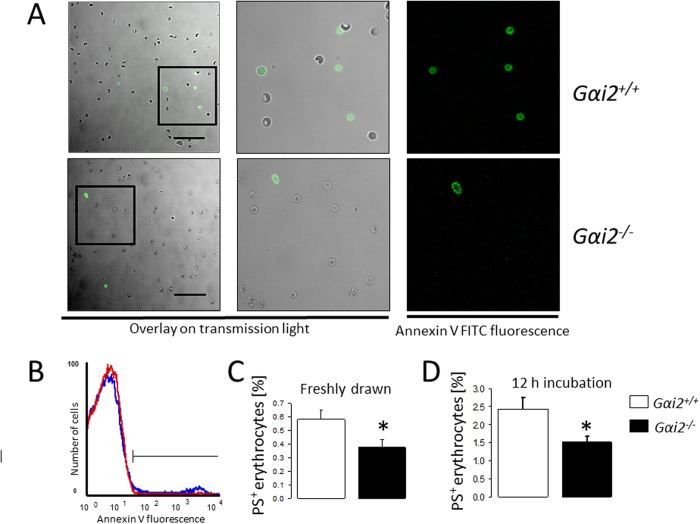
Phosphatidylserine externalization. (**A**) Confocal microscopy of annexin-V-fluorescence (right panels) and transmission light (middle and left panels) of erythrocytes from *Gαi2*^+/+^ and *Gαi2*^−/−^ mice. Middle panels are amplified images of the area inside the squares of left panels. (**B**) Histogram (*Blue: Gαi2*^+/+^*, red: Gαi2*^−/−^) and means ± SEM of annexin-V-binding in erythrocytes freshly drawn (**C,** n = 24–40) or incubated 12 h in Ringer (**D**, n = 11–17). *(p < 0.05) significantly different from *Gαi2*^+/+^ mice.

**Figure 4:**
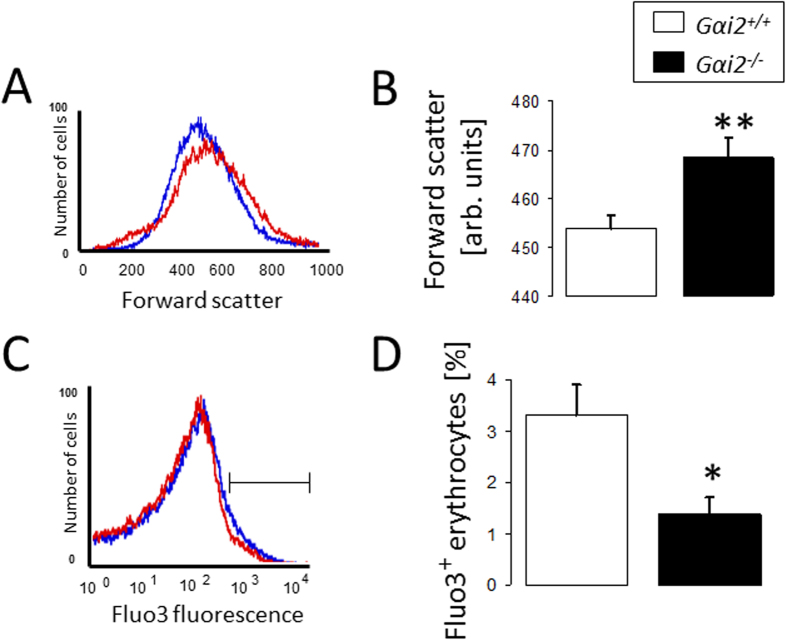
Cell shrinkage and cytosolic Ca^2+^-activity. Histogram (**A,C***; Blue: Gαi2*^+/+^*, red: Gαi2*^−/−^) and means ± SEM of forward scatter (**B**, n = 21–33) and percentage of Fluo3 positive erythrocytes (**D**, n = 8–16). *^,^**(p < 0.05, p < 0.01) significantly different from *Gαi2*^+/+^ mice.

Next, we explored whether Gαi2 deficiency influences erythrocyte survival. To this end, using annexin V binding, forward scatter and Fluo3 fluorescence in FACS analysis we analyzed erythrocyte cell membrane PS exposure, cell shrinkage and cytosolic Ca^2+^ activity, respectively. As depicted in Fig. 3A, freshly drawn and untreated erythrocytes were visualized using confocal microscopy and quantification of multiple fields showed a decreased ratio of annexin V binding cells to total cells (observed under transmission light) per field in *Gαi2*^−/−^ erythrocytes (0.028 ± 0.007; n = 4) as compared to *Gαi2*^+/+^ erythrocytes (0.069 ± 0.007; n = 4). PS exposure was simultaneously quantified using FACS analysis (50,000 cells were quantified) and confirmed that in both freshly drawn blood (Fig. 3B,C) and following 12 h incubation in Ringer solution (Fig. 3D), the percentage of annexin V binding erythrocytes was significantly lower in *Gαi2*^−/−^ mice than in *Gαi2*^+/+^ mice. Quantification of forward scatter showed that the cell volume was significantly larger in *Gαi2*^−/−^ erythrocytes as compared to *Gαi2*^+/+^ erythrocytes (Fig. 4A,B). Both cell membrane PS exposure and cell shrinkage are influenced by cytosolic Ca^2+^ activity^20^. As shown in Fig. 4C,D, the percentage of Fluo3 positive erythrocytes was slightly but significantly lower in *Gαi2*^−/−^ mice as compared to *Gαi2*^+/+^ mice. These data suggest an inhibitory effect of Gαi2 deficiency on eryptosis.

**Figure 5:**
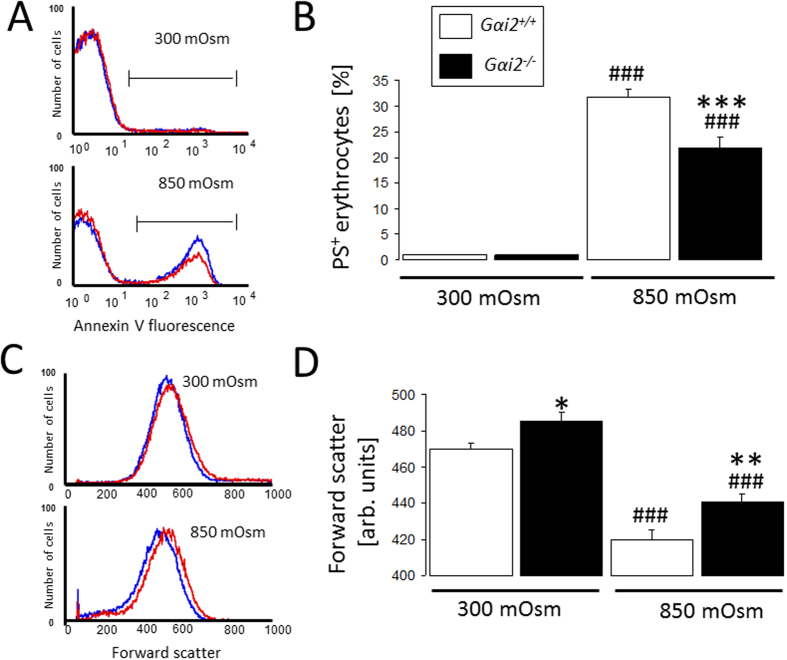
Effect of hyperosmolarity on phosphatidylserine externalization and cell shrinkage. Histogram (**A,C**; *Blue: Gαi2*^+/+^, *red: Gαi2*^−/−^) and means ± SEM of annexin-V-binding (**B**, n = 11–14) and forward scatter (**D**, n = 11–14) following 30 min incubation in isosmotic (300 mOsm) or hyperosmotic (850 m Osm) Ringer. ^###^(p < 0.001) significantly different from isosmotic, *^,^**^,^***(p < 0.05, p < 0.01, p < 0.001) from *Gαi2*^+/+^.

Further experiments then addressed the susceptibility of Gαi2-deficient erythrocytes to osmotic shock *ex vivo*, a pathophysiological cell stressor and a known stimulator of eryptosis. As illustrated in Fig. 5A,B, exposure of erythrocytes for 30 min to hyperosmotic Ringer (550 mM sucrose was added to the Ringer solution to reach the final osmolarity of 850 mOsm), significantly enhanced PS exposure, an effect, however, significantly blunted in *Gαi2*^−/−^ erythrocytes as compared to *Gαi2*^+/+^ erythrocytes. Erythrocyte forward scatter was quantified to determine hyperosmotic shock-triggered cell shrinkage. As shown in Fig. 5C,D, forward scatter was significantly reduced by hyperosmotic shock in erythrocytes from both *Gαi2*^−/−^ and *Gαi2*^+/+^ mice. The effect was significantly less pronounced in *Gαi2*^−/−^ erythrocytes than in *Gαi2*^+/+^ erythrocytes.

**Figure 6:**
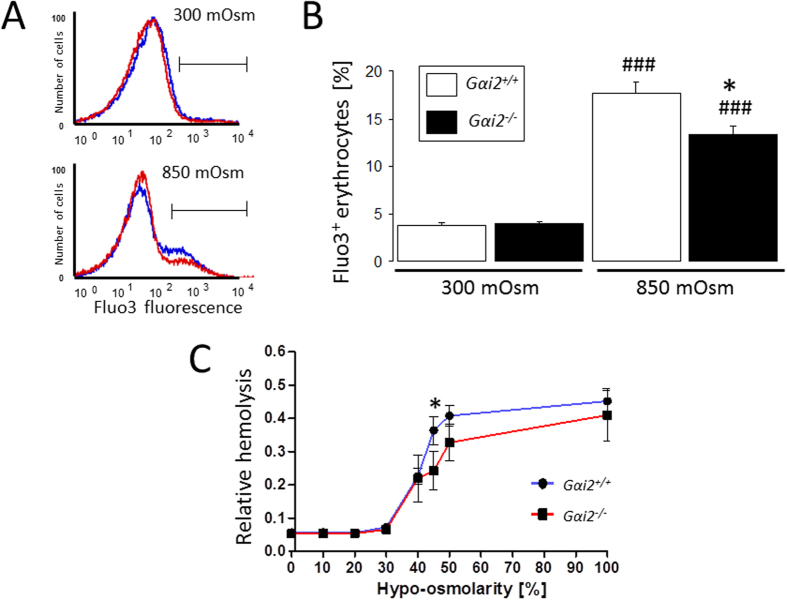
Effect of osmotic changes on cytosolic Ca^2+^-activity and hemolysis. Histogram (**A**; *Blue: Gαi2*^+/+^, *red: Gαi2*^−/−^) and means ± SEM of the percentage of erythrocytes with enhanced Fluo3-fluorescence (**B**, n = 11–14) following 30 min incubation in isosmotic (300 mOsm) or hyperosmotic (850 mOsm) Ringer. (**C**) Means ± SEM (n = 3–5) of relative hemolysis as a function of extracellular osmolarity (in % of isomotic Ringer) in *Gαi2*^+/+^ (*blue*) and *Gαi2*^−/−^ (*red*) erythrocytes. ^###^(p < 0.001) significantly different from isosmotic, *(p < 0.05) from *Gαi2*^+/+^.

To elucidate the mechanism contributing to the protective effect of Gαi2 deficiency against hyperosmotic shock-triggered eryptosis, we determined erythrocyte cytosolic Ca^2+^ activity following hyperosmotic shock. As shown in Fig. 6A,B, exposure of erythrocytes to hyperosmotic shock significantly enhanced the percentage of Fluo3 positive erythrocytes. The effect was, however, significantly blunted in *Gαi2*^−/−^ erythrocytes as compared to *Gαi2*^+/+^ erythrocytes. Further experiments explored the resistance of erythrocytes to a decline of extracellular osmolarity. As illustrated in Fig. 6C, the resistance of erythrocytes to graded decrease of osmolarity was significantly lower in Gαi2^+/+^ than in Gαi2^−/−^ erythrocytes. Thus, Gαi2 deficiency counteracts the sensitivity of erythrocytes to both hyper- and hypoosmotic shock.

**Figure 7:**
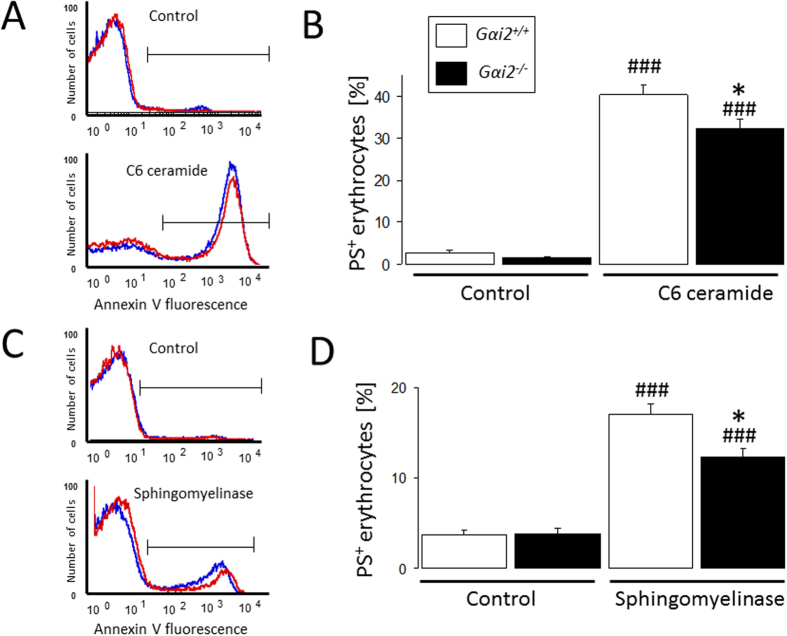
Effect of C6-ceramide and bacterial sphingomyelinase on phosphatidylserine externalization. Histograms (**A,C**; *Blue: Gαi2*^+/+^*, red: Gαi2*^−/−^) and means ± SEM of annexin-V-binding following exposure to C6-ceramide (**A,B**; 50 μM, 12 h; n = 11–17) or bacterial sphingomyelinase (**C,D**; 0.01 U/ml 24 h; n = 7–16). ^###^(p < 0.001) significantly different from Control. *(p < 0.05) from *Gαi2*^+/+^.

Additional experiments explored whether erythrocyte Gαi2 deficiency protects against ceramide-sensitive eryptosis. As shown in Fig. 7, treatment of erythrocytes from *Gαi2*^−/−^ and *Gαi2*^+/+^ mice with C6 ceramide and bacterial sphingomyelinase significantly increased PS exposure, an effect, slightly, but significantly less pronounced in *Gαi2*^−/−^ erythrocytes as compared to *Gαi2*^+/+^ erythrocytes. Thus, erythrocyte Gαi2 deficiency has a subtle effect on ceramide-elicited eryptosis.

## Discussion

The present observations disclose the expression of Gαi2 in human and murine erythrocytes and further reveals that Gαi2 deficiency confers partial protection against suicidal erythrocyte death or eryptosis. Our findings show that the percentage of eryptotic cells in circulating blood is significantly lower in *Gαi2*^−/−^ mice as in *Gαi2*^+/+^ mice. *Gαi2*^−/−^ mice do not show overt changes in erythrocyte parameters such as erythrocyte count, hematocrit, hemoglobin concentration and reticulocyte count. The impact of Gαi2 deficiency on erythrocytes is unmasked in the presence of pathophysiological cell stressors *ex vivo* such as hyperosmotic shock and following treatment with C6 ceramide and bacterial sphingomyelinase, whereby eryptosis is significantly less pronounced in *Gαi2*^−/−^ erythrocytes as compared to *Gαi2*^+/+^ erythrocytes.

Our data show that in the absence of stress, the difference between the percentage of PS-exposing erythrocytes in *Gαi2*^+/+^ mice and *Gαi2*^−/−^ mice is subtle (~0.2%) yet statistically significant. Previous studies have shown that spontaneous PS exposure in freshly drawn erythrocytes from healthy wild-type mice of different strains does not exceed 1%^19^ of the total number of circulating erythrocytes. Thus, in transgenic mice which display a phenotype of reduced eryptosis, the percentage of PS-exposing circulating erythrocytes may be significantly lower than in wild-type mice despite relatively lower magnitudes of difference. Exposure of erythrocytes to hypertonic extracellular environment *in vitro* simulates the osmotic conditions encountered in the kidney medulla^20^. In conditions such as acute renal failure, erythrocytes may enter eryptosis due to their entrapment in the kidney medulla^21^. Gαi2 deficiency may blunt eryptosis and thus favorably influence the respective clinical condition. Our data show that, in addition to curtailing PS exposure, *Gαi2*^−/−^ erythrocytes showed increased resistance to cell shrinkage following hyperosmotic shock. Accordingly, the mean corpuscular cell volume was significantly larger in *Gαi2*^−/−^ erythrocytes. Along those lines, it is intriguing to speculate that Gαi2 influences cell volume regulatory ion channels in erythrocytes.

Mechanistically, hyperosmotic shock is a powerful stimulator of Ca^2+^ entry and ceramide formation in erythrocytes^20^. We observed that following hyperosmotic shock of erythrocytes, Gαi2 deficiency leads to subtle but significant decrease of cytosolic Ca^2+^ entry. On the other hand, Gαi2 may additionally mediate hyperosmotic shock-induced eryptosis by influencing ceramide signaling^21^. This is corroborated by our data showing a mitigating effect of Gαi2 deficiency on eryptosis triggered by either C6 ceramide or bacterial sphingomyelinase. Ceramide sensitizes erythrocytes to the eryptotic effect of enhanced Ca^2+^ concentration and may stimulate eryptosis without appreciable increase in cytosolic Ca^2+^ activity^27^. Ceramide further modifies the interaction of the erythrocyte membrane with the cytoskeleton thereby increasing membrane fragility^28^. As Gαi2 is an essential regulator for Ca^2+^ signaling in nucleated cells, it is possible that the inhibitory effect of Gαi2 deficiency on erythrocyte death is, at least in part, mediated by its influence on cytosolic Ca^2+^ activity.

Eryptosis is inhibited by catecholamines including dopamine^29^. Interestingly, dopamine-dependent signaling involves pertussis toxin-sensitive Gαi2^30^. Further signaling molecules that regulate the eryptosis machinery include AMPK^20^, p38 MAPK^31^, CK1α^32^, PAK2^33^, PDK1^20^, MSK1/2^34^ and CDK4^35^. Eryptosis is triggered by a myriad of xenobiotics and endogenous substances^20^,^36^,^37^,^38^,^39^,^40^,^41^,^42^,^43^,^44^,^45^,^46^,^47^,^48^, and accelerated eryptosis contributes to the anemia associated with several clinical disorders^20^, including iron deficiency^49^, sepsis^50^, renal failure^51^, hepatic failure^52^, malignancy^24^, ageing^53^ and Wilson’s disease^54^. Eryptotic erythrocytes adhere to the vascular wall^55^, and stimulate blood clotting^56^. Excessive eryptosis may thus interfere with microcirculation and participate in the vascular injury of metabolic syndrome^57^. Accordingly, *Gαi2*^−/−^ mice may be particularly resistant to derangements of microcirculation following exposure to triggers of eryptosis. Moreover, eryptosis has been shown to influence the quality of stored erythrocytes^58^. Pharmacologically targeting Gαi2, at least in theory, may further provide new avenues in the treatment of conditions associated with anemia resulting from increased eryptosis^20^. On the other hand, Gαi2 modulation may serve as a novel target for the treatment of malaria, a condition where eryptosis plays a protective role in ameliorating parasitemia by expediting the clearance of pathogen-infected erythrocytes^20^.

In conclusion, the G-protein subunit Gαi2 is expressed in human and murine erythrocytes and participates in the regulation of erythrocyte suicide.

## Materials and Methods

### Mice

Experiments were performed in Gαi2 knockout mice *(Gαi2*^−/−^) and their wild type littermates (*Gαi2*^+/+^) of 6–9 weeks of age. The mice were generated and initially characterized on a SV129 background^18^. Mice were backcrossed on a C57BL6 background and kept under specified pathogen-free (SPF) environment in individually ventilated cages (IVC) to prolong life expectancy^4^,^59^. All animal experiments were conducted according to the German law for the care and use of laboratory animals and were approved by local government authorities (Regierungspräsidium Tübingen).

### Blood count, incubation and solutions

For all experiments except for the blood count, heparin blood was retrieved from the retrobulbar plexus of mice. For the blood count, EDTA blood was analyzed using an electronic hematology particle counter (type MDM 905 from Medical Diagnostics Marx; Butzbach, Germany) equipped with a photometric unit for haemoglobin determination. Plasma erythropoietin levels were determined using an immunoassay kit according to the manufacturer’s instructions (R&D Systems, Wiesbaden, Germany). Murine erythrocytes were isolated by being washed two times with Ringer solution containing (in mM): 125 NaCl, 5 KCl, 1 MgSO_4_, and 32 HEPES/NaOH (pH 7.4), 5 glucose, and 1 CaCl_2_. Where indicated, sucrose (550 mM), C6 ceramide (50 μM; Sigma) or bacterial sphingomyelinase (0.01 U/ml; Sigma) were added to the Ringer solution. May-Grünwald staining was used to examine changes in erythrocyte shape. Briefly, 20 μl of erythrocytes were smeared and fixed using methanol onto a glass slide, and stained with 5% Giemsa Azur-Eosin (Merck Millipore, Germany) in phosphate-buffered saline (in mM: 1.05 KH_2_PO_4_, 2.97 Na_2_HPO_4_, 155.2 NaCl) for 20 min. Subsequently, images were taken on a Nikon Diaphot 300 Microscope (Nikon Instruments, Germany).

### Reticulocyte count and markers of erythrocyte maturation

For determination of the reticulocyte count EDTA-whole blood (2.5 μl) was added to 500 μl Retic-COUNT (Thiazole orange) reagent from Becton Dickinson. Samples were stained for 30 min at room temperature, and flow cytometry was performed according to the manufacturer’s instructions. Forward scatter (FSC), side scatter (SSC), and thiazole orange-fluorescence intensity (in FL-1) of the blood cells were determined. The number of Retic-COUNT positive reticulocytes was expressed as the percentage of the total gated erythrocyte populations. Gating of erythrocytes was achieved by analysis of FSC vs. SSC dot plots using CellQuest software. To further examine the dynamic maturation of erythrocytes *in vivo*, erythrocytes were double stained with CD71 (1:12.5; BD Biosciences), and Ter119 (1:250; BD Biosciences). Ter119 and CD71 positive cells were quantified by analyzing the upper right quadrant of an FL1 versus FL2 dot plot.

### Phosphatidylserine exposure and forward scatter

After incubation, erythrocytes were washed once in Ringer solution containing 5 mM CaCl_2_. The cells were then stained with annexin-V-FITC (1:250 dilution; Immunotools, Friesoythe, Germany) at a 1:500 dilution. After 15 min, samples were measured by flow cytometric analysis (FACS-Calibur; BD). Cells were analyzed by forward scatter, and annexin V fluorescence intensity was measured in fluorescence channel FL-1 with excitation and emission wavelengths of 488 nm and 530 nm, respectively, on a FACS Calibur (BD, Heidelberg, Germany) as described previously^24^. Where indicated, spontaneous PS exposure and forward scatter were determined by addition of 2 μl of freshly drawn erythrocytes in 500 μl Ringer solution containing 5 mM CaCl_2_ and annexin-V-FITC. Raw data for annexin V positive erythrocytes was collected by a primary gating of the erythrocyte population on FSC vs. SSC dot plots and, subsequently, by setting an arbitrary marker at the base of the cell population on an FL1 histogram. The cell population plotted on the left of the arbitrary marker was considered positive for annexin V binding.

### Estimation of intracellular Ca^2+^

For measurement of intracellular Ca^2+^, 50 μl erythrocyte suspension was washed in Ringer solution and then loaded with Fluo-3/AM (Biotrend, Köln, Germany) in Ringer solution containing 5 mM CaCl_2_ and 5 μM Fluo-3/AM. The cells were incubated at 37 °C for 30 min and washed twice in Ringer solution containing 5 mM CaCl_2_. The Fluo-3/AM-loaded erythrocytes were resuspended in 200 μl Ringer. Then, Ca^2+^-dependent fluorescence intensity was measured in the fluorescence channel FL-1 in FACS analysis. Where indicated, spontaneous intracellular Ca^2+^ was determined by addition of 2 μl of freshly drawn erythrocytes in 500 μl Ringer solution containing 5 mM CaCl_2_ as well as Fluo3/AM. Fluo3 positive cells were plotted using an FL1 histogram similar to the analysis of annexin V positive cells.

### Determination of the osmotic resistance

Two microliters of blood were added to 200 μl of PBS solutions with decreasing osmolarity. After centrifugation for 5 min at 3000 rpm, the supernatant was transferred to a 96-well plate, and the absorption at 405 nm was determined as a measure of hemolysis. Absorption of the supernatant of erythrocytes lysed in pure distilled water was defined as 100% hemolysis.

### Immunoblotting

To examine the expression of Gαi2 in human or murine erythrocytes, 150 μl erythrocyte pellet was lysed in 50 ml of 20 mM HEPES/NaOH (pH 7.4). Ghost membranes were pelleted (15,000 g for 20 min at 4 °C) and lysed in 200 μl lysis buffer (50 mM Tris-HCl, pH 7.5; 150 mM NaCl; 1% Triton X-100; 0.5% SDS; 1 mM NaF; 1 mM Na_3_VO_4_; and 0.4% β-mercaptoethanol) containing protease inhibitor cocktail (Sigma, Schnelldorf, Germany). Triton X-100, a non-ionic detergent, was used in erythrocyte ghost preparation due to its effective solubilization power and a relatively mild effect on membrane-bound enzymes^60^. In all cases, 60 μg of protein was solubilized in Laemmli sample buffer at 95 °C for 5 min and resolved by pre-casted 10% SDS-PAGE gel (Invitrogen, Karlsruhe, Germany). For immunoblotting, proteins were electrotransferred onto a polyvinylidene difluoride (PVDF) membrane and blocked with 5% nonfat milk in TBS-0.10% Tween 20 at room temperature for 1 h. Then, the membrane was incubated with anti-G-protein alpha inhibitor 2 antibody (1:5000; 40 kDa; Abcam Cat# ab157204) at 4 °C overnight. After being washed (in TBS-0.10% Tween 20) and subsequently blocked, the blots were incubated with secondary anti-rabbit antibody (1:2000; Cell Signaling) for 1 h at room temperature. After being washed, the antibody binding was detected with the ECL detection reagent (Amersham, Freiburg, Germany).

### Confocal microscopy and immunofluorescence

For the visualization of eryptotic erythrocytes, 4 μl of erythrocytes, incubated in the respective experimental solutions, were stained with FITC-conjugated annexin-V (1:100 dilution; ImmunoTools, Friesoythe, Germany) in 200 μl Ringer solution containing 5 mM CaCl_2_. Then, the erythrocytes were washed twice and finally resuspended in 50 μl of Ringer solution containing 5 mM CaCl_2_. Twenty μl were mounted with Prolong Gold antifade reagent (Invitrogen, Darmstadt, Germany) onto a glass slide and covered with a coverslip. Sections were analyzed using a Leica TCS-SP / Leica DM RB confocal laser scanning microscope. Images were processed with Leica Confocal Software LCS (Version 2.61).

### Statistics

Data are expressed as arithmetic means ± SEM, and statistical analysis was made using ANOVA or *t*-test, as appropriate. n denotes the number of different erythrocyte specimens studied.

## Additional Information

**How to cite this article**: Bissinger, R. *et al.* Blunted apoptosis of erythrocytes in mice deficient in the heterotrimeric G-protein subunit Gαi2. *Sci. Rep.* **6**, 30925; doi: 10.1038/srep30925 (2016).

## References

[b1] Carmichael C. Y. & Wainford R. D. Brain Galphai 2 -subunit proteins and the prevention of salt sensitive hypertension. Front Physiol 6, 233 (2015). 26347659 10.3389/fphys.2015.00233 PMC4541027

[b2] Wettschureck N. & Offermanns S. Mammalian G proteins and their cell type specific functions. Physiol Rev 85, 1159–1204 (2005). 16183910 10.1152/physrev.00003.2005

[b3] Simon M. I., Strathmann M. P. & Gautam N. Diversity of G proteins in signal transduction. Science 252, 802–808 (1991). 1902986 10.1126/science.1902986

[b4] Devanathan V. *et al.* Platelet Gi protein Galphai2 is an essential mediator of thrombo-inflammatory organ damage in mice. Proc. Natl. Acad. Sci. USA 112, 6491–6496 (2015). 25944935 10.1073/pnas.1505887112 PMC4443332

[b5] Pero R. S. *et al.* Galphai2-mediated signaling events in the endothelium are involved in controlling leukocyte extravasation. Proc. Natl. Acad. Sci. USA 104, 4371–4376 (2007). 17360531 10.1073/pnas.0700185104 PMC1838609

[b6] Ngai J., Inngjerdingen M., Berge T. & Tasken K. Interplay between the heterotrimeric G-protein subunits Galphaq and Galphai2 sets the threshold for chemotaxis and TCR activation. BMC. Immunol 10, 27 (2009). 19426503 10.1186/1471-2172-10-27 PMC2694176

[b7] Zarbock A., Deem T. L., Burcin T. L. & Ley K. Galphai2 is required for chemokine-induced neutrophil arrest. Blood 110, 3773–3779 (2007). 17699741 10.1182/blood-2007-06-094565 PMC2077322

[b8] Minetti G. C. *et al.* Galphai2 signaling is required for skeletal muscle growth, regeneration, and satellite cell proliferation and differentiation. Mol. Cell Biol 34, 619–630 (2014). 24298018 10.1128/MCB.00957-13 PMC3911486

[b9] Lopez-Aranda M. F. *et al.* Activation of caspase-3 pathway by expression of sGalphai2 protein in BHK cells. Neurosci. Lett 439, 37–41 (2008). 18502580 10.1016/j.neulet.2008.04.078

[b10] Myeong J. *et al.* Close spatio-association of the transient receptor potential canonical 4 (TRPC4) channel with Galphai in TRPC4 activation process. Am. J. Physiol Cell Physiol 308, C879–C889 (2015). 25788576 10.1152/ajpcell.00374.2014

[b11] Dizayee S. *et al.* Galphai2- and Galphai3-specific regulation of voltage-dependent L-type calcium channels in cardiomyocytes. PLoS. One 6, e24979 (2011). 21966394 10.1371/journal.pone.0024979 PMC3180279

[b12] Dezaki K., Kakei M. & Yada T. Ghrelin uses Galphai2 and activates voltage-dependent K+ channels to attenuate glucose-induced Ca2+ signaling and insulin release in islet beta-cells: novel signal transduction of ghrelin. Diabetes 56, 2319–2327 (2007). 17575083 10.2337/db07-0345

[b13] Cao C. *et al.* Galpha(i1) and Galpha(i3) are required for epidermal growth factor-mediated activation of the Akt-mTORC1 pathway. Sci. Signal 2, ra17 (2009). 19401591 10.1126/scisignal.2000118 PMC4138699

[b14] Duronio V. The life of a cell: apoptosis regulation by the PI3K/PKB pathway. Biochem. J 415, 333–344 (2008). 18842113 10.1042/BJ20081056

[b15] Jeon J. P. *et al.* Activation of TRPC4beta by Galphai subunit increases Ca2+ selectivity and controls neurite morphogenesis in cultured hippocampal neuron. Cell Calcium 54, 307–319 (2013). 24011658 10.1016/j.ceca.2013.07.006

[b16] Singh V., Raghuwanshi S. K., Smith N., Rivers E. J. & Richardson R. M. G Protein-coupled receptor kinase-6 interacts with activator of G protein signaling-3 to regulate CXCR2-mediated cellular functions. J Immunol 192, 2186–2194 (2014). 24510965 10.4049/jimmunol.1301875 PMC3950967

[b17] Zuberi Z. *et al.* Absence of the inhibitory G-protein Galphai2 predisposes to ventricular cardiac arrhythmia. Circ Arrhythm Electrophysiol 3, 391–400 (2010). 20495013 10.1161/CIRCEP.109.894329 PMC3401367

[b18] Rudolph U. *et al.* Ulcerative colitis and adenocarcinoma of the colon in G alpha i2-deficient mice. Nat. Genet 10, 143–150 (1995). 7663509 10.1038/ng0695-143

[b19] Lang F. & Qadri S. M. Mechanisms and significance of eryptosis, the suicidal death of erythrocytes. Blood Purif 33, 125–130 (2012). 22269222 10.1159/000334163

[b20] Lang E., Qadri S. M. & Lang F. Killing me softly-suicidal erythrocyte death. Int. J. Biochem. Cell Biol 44, 1236–1243 (2012). 22561748 10.1016/j.biocel.2012.04.019

[b21] Lang K. S. *et al.* Involvement of ceramide in hyperosmotic shock-induced death of erythrocytes. Cell Death. Differ 11, 231–243 (2004). 14615798 10.1038/sj.cdd.4401311

[b22] Foller M. *et al.* TRPC6 contributes to the Ca(2+) leak of human erythrocytes. Cell Physiol Biochem 21, 183–192 (2008). 18209485 10.1159/000113760

[b23] Zidova Z. *et al.* DMT1-mutant erythrocytes have shortened life span, accelerated glycolysis and increased oxidative stress. Cell Physiol Biochem 34, 2221–2231 (2014). 25562168 10.1159/000369665

[b24] Qadri S. M. *et al.* Enhanced suicidal erythrocyte death in mice carrying a loss-of-function mutation of the adenomatous polyposis coli gene. J. Cell Mol. Med 16, 1085–1093 (2012). 21781276 10.1111/j.1582-4934.2011.01387.x PMC4365887

[b25] Foller M. *et al.* Functional significance of glutamate-cysteine ligase modifier for erythrocyte survival *in vitro* and *in vivo*. Cell Death. Differ 20, 1350–1358 (2013). 23787995 10.1038/cdd.2013.70 PMC3770325

[b26] Ohman L., Franzen L., Rudolph U., Harriman G. R. & Hultgren H. E. Immune activation in the intestinal mucosa before the onset of colitis in Galphai2-deficient mice. Scand. J. Immunol 52, 80–90 (2000). 10886787 10.1046/j.1365-3083.2000.00759.x

[b27] Lang E., Bissinger R., Gulbins E. & Lang F. Ceramide in the regulation of eryptosis, the suicidal erythrocyte death. Apoptosis 20, 758–767 (2015). 25637185 10.1007/s10495-015-1094-4

[b28] Dinkla S. *et al.* Functional consequences of sphingomyelinase-induced changes in erythrocyte membrane structure. Cell Death. Dis 3, e410 (2012). 23076218 10.1038/cddis.2012.143 PMC3481131

[b29] Lang P. A. *et al.* Inhibition of erythrocyte “apoptosis” by catecholamines. Naunyn Schmiedebergs Arch. Pharmacol 372, 228–235 (2005). 16247607 10.1007/s00210-005-0009-2

[b30] Neve K. A., Seamans J. K. & Trantham-Davidson H. Dopamine receptor signaling. J. Recept. Signal. Transduct. Res 24, 165–205 (2004). 15521361 10.1081/rrs-200029981

[b31] Gatidis S. *et al.* p38 MAPK activation and function following osmotic shock of erythrocytes. Cell Physiol Biochem 28, 1279–1286 (2011). 22179015 10.1159/000335859

[b32] Zelenak C. *et al.* Protein kinase CK1alpha regulates erythrocyte survival. Cell Physiol Biochem 29, 171–180 (2012). 22415086 10.1159/000337598

[b33] Zelenak C. *et al.* Proteome analysis of erythrocytes lacking AMP-activated protein kinase reveals a role of PAK2 kinase in eryptosis. J. Proteome. Res 10, 1690–1697 (2011). 21214270 10.1021/pr101004j

[b34] Lang E. *et al.* Accelerated apoptotic death and *in vivo* turnover of erythrocytes in mice lacking functional mitogen- and stress-activated kinase MSK1/2. Sci. Rep 5, 17316 (2015). 26611568 10.1038/srep17316 PMC4661433

[b35] Lang E. *et al.* Impact of Cyclin-Dependent Kinase CDK4 Inhibition on Eryptosis. Cell Physiol Biochem 37, 1178–1186 (2015). 26418250 10.1159/000430241

[b36] Arnold M., Bissinger R. & Lang F. Mitoxantrone-induced suicidal erythrocyte death. Cell Physiol Biochem 34, 1756–1767 (2014). 25427644 10.1159/000366376

[b37] Bissinger R., Fischer S., Jilani K. & Lang F. Stimulation of erythrocyte death by phloretin. Cell Physiol Biochem 34, 2256–2265 (2014). 25562171 10.1159/000369668

[b38] Bissinger R., Lupescu A., Zelenak C., Jilani K. & Lang F. Stimulation of eryptosis by cryptotanshinone. Cell Physiol Biochem 34, 432–442 (2014). 25095724 10.1159/000363012

[b39] Zhang R. *et al.* Involvement of calcium, reactive oxygen species, and ATP in hexavalent chromium-induced damage in red blood cells. Cell Physiol Biochem 34, 1780–1791 (2014). 25427846 10.1159/000366378

[b40] Tesoriere L. *et al.* Oxysterol mixture in hypercholesterolemia-relevant proportion causes oxidative stress-dependent eryptosis. Cell Physiol Biochem 34, 1075–1089 (2014). 25228229 10.1159/000366322

[b41] Risso A., Ciana A., Achilli C. & Minetti G. Survival and senescence of human young red cells *in vitro*. Cell Physiol Biochem 34, 1038–1049 (2014). 25227963 10.1159/000366319

[b42] Lupescu A., Bissinger R., Warsi J., Jilani K. & Lang F. Stimulation of erythrocyte cell membrane scrambling by gedunin. Cell Physiol Biochem 33, 1838–1848 (2014). 24969439 10.1159/000362962

[b43] Faggio C., Alzoubi K., Calabro S. & Lang F. Stimulation of suicidal erythrocyte death by PRIMA-1. Cell Physiol Biochem 35, 529–540 (2015). 25614142 10.1159/000369717

[b44] Peter T. *et al.* Programmed erythrocyte death following *in vitro* Treosulfan treatment. Cell Physiol Biochem 35, 1372–1380 (2015). 25720583 10.1159/000373958

[b45] Officioso A., Alzoubi K., Manna C. & Lang F. Clofazimine Induced Suicidal Death of Human Erythrocytes. Cell Physiol Biochem 37, 331–341 (2015). 26316080 10.1159/000430357

[b46] Fazio A., Briglia M., Faggio C., Alzoubi K. & Lang F. Stimulation of Suicidal Erythrocyte Death by Garcinol. Cell Physiol Biochem 37, 805–815 (2015). 26356270 10.1159/000430397

[b47] Lang E. *et al.* Vitamin D-Rich Diet in Mice Modulates Erythrocyte Survival. Kidney Blood Press Res 40, 403–412 (2015). 26227001 10.1159/000368517

[b48] Ran Q. *et al.* Eryptosis Indices as a Novel Predictive Parameter for Biocompatibility of Fe3O4 Magnetic Nanoparticles on Erythrocytes. Sci. Rep 5, 16209 (2015). 26537855 10.1038/srep16209 PMC4633654

[b49] Kempe D. S. *et al.* Enhanced programmed cell death of iron-deficient erythrocytes. FASEB J 20, 368–370 (2006). 16371427 10.1096/fj.05-4872fje

[b50] Kempe D. S. *et al.* Suicidal erythrocyte death in sepsis. J. Mol. Med. (Berl) 85, 273–281 (2007). 17180345 10.1007/s00109-006-0123-8

[b51] Abed M. *et al.* Suicidal erythrocyte death in end-stage renal disease. J. Mol. Med. (Berl) 92, 871–879 (2014). 24743961 10.1007/s00109-014-1151-4

[b52] Lang E. *et al.* Conjugated bilirubin triggers anemia by inducing erythrocyte death. Hepatology 61, 275–284 (2015). 25065608 10.1002/hep.27338 PMC4303990

[b53] Lupescu A. *et al.* Enhanced suicidal erythrocyte death contributing to anemia in the elderly. Cell Physiol Biochem 36, 773–783 (2015). 26021265 10.1159/000430137

[b54] Lang P. A. *et al.* Liver cell death and anemia in Wilson disease involve acid sphingomyelinase and ceramide. Nat. Med 13, 164–170 (2007). 17259995 10.1038/nm1539

[b55] Borst O. *et al.* Dynamic adhesion of eryptotic erythrocytes to endothelial cells via CXCL16/SR-PSOX. Am. J. Physiol Cell Physiol 302, C644–C651 (2012). 22173866 10.1152/ajpcell.00340.2011

[b56] Chung S. M. *et al.* Lysophosphatidic acid induces thrombogenic activity through phosphatidylserine exposure and procoagulant microvesicle generation in human erythrocytes. Arterioscler. Thromb. Vasc. Biol 27, 414–421 (2007). 17110600 10.1161/01.ATV.0000252898.48084.6a

[b57] Zappulla D. Environmental stress, erythrocyte dysfunctions, inflammation, and the metabolic syndrome: adaptations to CO2 increases? J. Cardiometab. Syndr 3, 30–34 (2008). 18326983 10.1111/j.1559-4572.2008.07263.x

[b58] Antonelou M. H., Kriebardis A. G. & Papassideri I. S. Aging and death signalling in mature red cells: from basic science to transfusion practice. Blood Transfus 8 Suppl 3, s39–s47 (2010). 20606748 10.2450/2010.007S PMC2897187

[b59] Wiege K. *et al.* Galphai2 is the essential Galphai protein in immune complex-induced lung disease. J. Immunol 190, 324–333 (2013). 23225882 10.4049/jimmunol.1201398

[b60] Helenius A. & Simons K. Solubilization of membranes by detergents. Biochim Biophys Acta 415, 29–79 (1975). 1091302 10.1016/0304-4157(75)90016-7

